# On the use of pseudoword reading as estimate of premorbid intelligence in brain injured, psychopathological, or cognitively impaired patients

**DOI:** 10.3389/fpsyg.2022.1048237

**Published:** 2023-01-04

**Authors:** Miguel Ángel Pérez-Sánchez, Javier Marín

**Affiliations:** Departamento de Psicología Básica y Metodología, Universidad de Murcia, Murcia, Spain

**Keywords:** intelligence quotient (IQ), reading, pseudoword, premorbid intelligence quotient, normative data, orthographic transparency

## Introduction

In this opinion article we discuss the adequacy to employ pseudoword reading (PWR) performance as an estimator of premorbid intelligence (PI), at least in transparent languages. Patients' current PWR level and scores on other reading tasks (e.g., irregular words reading) have been proposed as valid measures to estimate PI on the premises that they correlate with intelligence scores and show relative resistance to cognitive impairment caused by brain injury or illness (Del Pino et al., 2018, 2020). Del Pino et al. (2018) developed norms of behavioral data from two reading tasks (i.e., word accentuation and PWR) in adult Spanish speaking population in order to use them as estimates of PI in brain injured, psychopathological, or cognitively impaired populations. Later, Del Pino et al. (2020) suggested that given reading tasks could usefully complement other variables such as age and years of education to estimate more accurately the cognitive performance in Parkinson's disease. However, we defend the position that there is not sufficient direct evidence of the relationship between PWR and PI. In what follows, we provide arguments that challenge the use of PWR performance as an estimator of PI.

## Lack of substantive and consistent evidence of the validity of PWR as a predictor of PI

Let us first examine the arguments and evidence provided by those who hold that PWR is an adequate estimator of PI. Del Pino et al. (2018) claimed that “…reading ability becomes, with practice, an automatic ability that is highly resistant to cognitive impairment (sic) (Del Ser et al., 1997; Russell et al., 2000; Khandaker et al., 2011; Harman-Smith et al., 2013; Hessler et al., 2013). Hence, instruments based on reading irregular words (i.e., NART) (Nelson and Willison, 1991) or based on pseudo-words (PW) (i.e., Spot-the-Word test) (Baddeley et al., 1993) are used to estimate premorbid IQ.” (p. 2). Furthermore, Del Pino et al. (2018) also added that “concerning reading PW tests, the Spot-the-Word test (Baddeley et al., 1993) was proposed as an adequate instrument to assess premorbid IQ in older adults with normal aging as well as in patients with dementia (Friedman et al., 1992; Patterson et al., 1994; McFarlane et al., 2006).” (p. 2; see also Del Pino et al., 2020, for a similar argument). However, most of the cited investigations did not address the PWR task. Del Ser et al. (1997) examined the ability of Spanish speakers to stress the correct syllable in a series of unfamiliar words. Russell et al. (2000) investigated the validity of an irregular English words reading test (i.e., the National Adult Reading Test, NART; Nelson and Willison, 1991) as an estimate of PI in schizophrenia. Khandaker et al. (2011) meta-analysis examined the association between PI and the schizophrenia disorder, but no measurement of PWR was mentioned. Harman-Smith et al. (2013) showed that performance in a reading task of irregular English words is a valid estimation of PI in patients with brain injury. Hessler et al. (2013) investigated the feasibility of a standardized multiple choice vocabulary test (i.e., the MWT-B, Lehrl, 1999) to estimate PI in cognitive impairment. McFarlane et al. (2006) assessed the validity of several word-reading tests and a demographic regression equation in estimating PI in AD patients, but not the role of PWR. And importantly, the Spot-the-Word (STW) test is not a PWR task but a lexical decision task (i.e., participants must identify the word in a pair of items comprising one word and one pseudoword). Lastly, the two following studies did address PWR performance in patients with Alzheimer's disease (AD). On the one hand, Friedman et al. (1992) demonstrated that the ability to read aloud a specific kind of unfamiliar pseudowords remains relatively preserved in AD. The patients showed a poorer performance with the pseudowords that can be decoded exclusively on grapheme-phoneme correspondence (GPC) rules in comparison with the pseudowords that can also be read by analogy to alike words. In the light of these results, Friedman et al. concluded that in the frame of dual-route reading models (e.g., Coltheart et al., 2001), AD patients are successful in PWR by developing automatic lexical-analogy mechanisms based on the lexical reading route and not through the GPC route. On the other hand, Patterson et al. (1994) observed that PWR performance, in terms of number of reading errors, gradually declined across subgroups of AD at different (growing) levels of severity, even though all the employed items had conventional spelling patterns and rather word-like pronunciations (i.e., regular pseudowords). Moreover, most of errors made by the patients in the PWR task were lexicalization errors, which suggests that the patients were in fact relying on the lexical route. Thus, Patterson et al. suggested as a possible explanation that in AD there is partial damage “…in the ability to perform phonological manipulations such as segmentation and blending, which is particularly critical to reading of unfamiliar non-words.” (p. 406). In summary, the mentioned studies do not provide data related to the PWR task as an estimate of PI, while they do so for other word reading or vocabulary tasks, with mixed results.

But is there any evidence associating execution in PRW with intelligence measures? Table 1 summarizes the bivariate correlations and their confidence intervals found in nine previous studies between PRW and intelligence or cognitive domain measures in different samples of participants. Overall, we can observe mixed results, where the correlation sizes vary, in the classical terms by Cohen (1988), from small to medium in three studies (Siegel, 1993; Canivez et al., 2014; Del Pino et al., 2020), from not significant to small correlations in other three studies (Van den Bos, 1998; Cotton and Crewther, 2009; Rowe et al., 2012), from not significant to large correlations for different subsamples of children in one work (Stanovich et al., 1984), and finally from medium to large correlations in one study in Greek (Simos et al., 2013). More evidence comes from the study by Jimenez et al. (2003), who followed a factorial design. They found an effect of the Full Scale-IQ (from WISC-R; Wechsler, 1989) on PWR times in both subsamples of 94 Spanish and 157 English-speaking Canadian children with reading difficulties. The observed effect was a slight trend toward a poorer PWR performance of the groups with the lowest Full Scale-IQ (i.e., IQ < 80) in comparison with other groups with higher IQ. However, when the data were examined by intelligence subscales, the results showed that Verbal-IQ influenced PWR only in English (i.e., similarly to that observed with the Full Scale-IQ), but not in Spanish. And no effects were found with Performance-IQ either in English or Spanish.

**Table 1:** Bivariate correlations found between intelligence scales/cognitive domains with PRW.

**Study**	**Language**	**Sample**	**Intelligence measure**	***r-*Pearson value**	**99% CI (lower, upper)**
Canivez et al. (2014)	English (Ireland)	1,014 children with learning difficulties (6-to-16 yo.)	FSIQ (WISC-IV^UK^)	0.28^a^ ^***^	0.20, 0.35
			VCI (WISC-IV^UK^)	0.24^a^ ^***^	0.16, 0.31
			PRI (WISC-IV^UK^)	0.20^a^ ^***^	0.12, 0.27
			WMI (WISC-IV^UK^)	0.38^a^ ^***^	0.31, 0.45
			PSI (WISC-IV^UK^)	0.10^a^ ^**^	0.02, 0.18
Cotton and Crewther (2009)	English (Australia)	126 healthy children (7-to-11 yo.)	PPVT	0.26^b^ ^*^	0.03, 0.46
			RPM-C	0.20^b^ ns	−0.03, 0.41
Del Pino et al. (2020)	Spanish (Spain)	39 PD patients and 162 healthy adult controls	SF (Animals & Supermarket)	0.36^c^ ^***^	0.19, 0.51
			Verbal Memory (HVLT R Recall)	0.20^c^ ^**^	0.02, 0.37
			Visual Memory (BVMT R Recall)	0.41^c^ ^***^	0.25, 0.55
			Executive Functions (TMT-B)	0.43^c^ ^***^	0.27, 0.57
Landi (2010)	English (USA)	928 healthy adults	RAPM	0.105^d^ ^**^	0.02, 0.19
Rowe et al. (2012)	English (USA)	84 healthy children (7-to-14 yo.)	FSIQ (WISC-IV)	−0.00^e^ ns	−0.28, 0.28
			VCI (WISC-IV)	0.01^e^ ns	−0.27, 0.29
			PRI (WISC-IV)	0.06^e^ ns	−0.22, 0.33
			WMI (WISC-IV)	0.15^e^ ns	−0.13, 0.41
			PSI (WISC-IV)	−0.19^e^ ns	−0.45, 0.09
			GAI (WISC-IV)	0.05^e^ ns	−0.23, 0.32
Siegel (1993)	English (Canada)	1,493 healthy and with learning disabilities children (7-to-16 yo.)	IQS	0.39^f^ ^***^	0.33, 0.45
Simos et al. (2013)	Greek (Greece)	386 healthy adults	WASI Block Design	0.37^g^ ^***^	0.25, 0.47
			WASI Vocabulary	0.68^g^ ^***^	0.60, 0.74
			PPVT	0.65^g^ ^***^	0.56, 0.72
Stanovich et al. (1984)	English (USA)	56 healthy children (1^st^ grade)	PPVT	−0.21^h^ ns	−0.51, 0.14
			RPM-SC	−0.15^h^ ns	−0.47, 0.20
		18 healthy children (3^rd^ grade)	PPVT	−0.01^h^ ns	−0.58, 0.57
			RPM-SC	0.04^h^ ns	−0.55, 0.60
		20 healthy children (5^th^ grade)	PPVT	−0.64^h^ ^**^	−0.88, −0.13
			RPM-SC	−0.70^h^ ^***^	−0.90, −0.24
Van den Bos (1998)	Dutch (Netherlands)	87 children with reading difficulties (10-to-12 yo.)	Intelligence factor	−0.27^i^ ^*^	−0.51, 0.00
			Intelligence factor	−0.27^j^ ^*^	−0.51, 0.00
			VSIQ (WISC-R^DU^)	−0.13^i^ ns	−0.39, 0.15
			VSIQ (WISC-R^DU^)	−0.09^j^ ns	−0.35, 0.19
			PSIQ (WISC-R^DU^)	−0.23^i^ ns	−0.47, 0.04
			PSIQ (WISC-R^DU^)	−0.15^j^ ns	−0.40, 0.13

WISC–IV^UK^ (Wechsler, 2004). FSIQ, Full Scale IQ. VCI, Verbal Comprehension Index; PRI, Perceptual Reasoning Index; WMI, Working Memory Index; PSI, Processing Speed Index. PPVT, Peabody Picture Vocabulary Test (Dunn and Dunn, 1965). RPM-C, Raven's Progressive Matrices, Colored (Raven et al., 1998). PD, Parkinson's disease. SF, semantic fluency (Schretlen and Vannorsdall, 2010). HVLT-R, Hopkins verbal learning Test-Revised (Brandt and Benedict, 2001). BVMT-R, Brief Visuospatial Memory Test-Revised (Schretlen et al., 1996). TMT B, Trail Making Test Part B (Horton and Hartlage, 1994). RAPM, Raven's Advanced Progressive Matrices (Raven and Raven, 2003). WISC–IV (Wechsler, 2003). GAI, General Ability Index. IQS, Intelligence Quotient Score taken from the Full Scale WISC-R, estimated WIS-R, or PPVT. WASI, Wechsler Abbreviated Scale of Intelligence (Wechsler, 1999). RPM-SC, Raven's Progressive Matrices, Standard and/or Colored (Raven, 1978). Intelligence factor, a factor composed of the scales of WISC–R^DU^ (Van Haasen et al., 1986) plus a measure of phonological awareness (rhyme and alliteration). VSIQ, Verbal Scale IQ. PSIQ, Performance Scale IQ.

a Pseudoword Decoding subtest from Wechsler Individual Achievement Test—Second UK Edition (Wechsler, 2005).

b Word Attack task.

c Pseudo-Words subtest form PROLEC-R (Cuetos et al., 2007).

d Pseudohomophone choice task, where participants have to read aloud pseudowords and answer whether or not they had the same pronunciation as a real word.

e Pseudoword Decoding subtest from Wechsler Individual Achievement Test (Wechsler, 2001).

f Combined percentile scores from Reading Symbols subtest of the GFW Sound-Symbol Test (Goldman et al., 1974) or the Word Attack subtest of the Woodcock Reading Mastery Test (Woodcock, 1973).

g Reading accuracy of list of 70 one- to six-syllable pseudowords.

h Mean naming time of 15 monosyllable pseudowords.

i Mean processing times per item, 2-min pseudoword reading test (Van den Bos et al., 1994).

j Percentage of errors, 2 min pseudoword reading test.

*** p < 0.001.

** p < 0.005.

* p < 0.01. ns, p > 0.01.

Most of these studies have examined the relationship between PWR and intelligence in children, employing sometimes scarce samples, or with mixed or special populations, which makes it difficult to generalize the results to adult healthy or general population. If we focus only on the studies with healthy children, there is a trend to an absence of connection (i.e., negligible to small correlations) between PWR and intelligence (Cotton and Crewther, 2009; Rowe et al., 2012), except that observed by Stanovich et al. (1984) in the healthy 5th-grade children subsample (*N* = 20). Unfortunately, in the studies by Siegel (1993) and Del Pino et al. (2020) the correlations were not reported separately for healthy and unhealthy subsamples, so it is not possible to know how much of these effects corresponds to each subsample. The only two studies that reported correlations for a healthy adult sample found that the correlation between PWR and non-verbal intelligence scores was statistically significantly but small (Landi, 2010; Simos et al., 2013), while the correlation between PWR and verbal intelligence scores was large (Simos et al., 2013).

Additionally, functional neuroimaging of brain networks underlying PWR evidences considerable independence of IQ from WASI (Wechsler, 1999). Simos et al. (2014) used magnetoencephalography to analyse the mediation of IQ in a PWR task. The participants in this study were 127 students from 6 to 14 years old with reading difficulties and 62 paired healthy controls. The authors found that the two hypoactivated areas of the left temporo-parietal cortex (i.e., the left superior temporal and supramarginal gyri) in the students experiencing reading difficulties was not affected by IQ. They also found in both samples of participants a positive association between phonological decoding ability (i.e., accuracy rate in the PWR task) and the degree of activation in the left fusiform gyrus and, importantly, given association was not modulated by IQ.

In sum, indirect and direct evidence, with the latter being very scarce, about the relationship between PRW and IQ provide mixed results, with most studies obtaining small or negligible effects.

## The orthographic transparency in decoding acquisition

The spelling system is another factor to consider in reading (e.g., Frost, 2012). Differential effects in reading acquisition and adult reading have been found for languages that vary in the transparency of their spelling and in their metric systems (e.g., Seymour et al., 2003). Cross-language studies show that decoding skills (i.e., the application of GPC rules to letter and pseudoword reading) are learnt relatively early and with ease by normal child readers of transparent orthographies, while in languages with opaque orthographies these skills are established slower. Additionally, individual variability in decoding is much greater in opaque than in transparent languages (Seymour et al., 2003; Seymour, 2005), the relevance of decoding decreases with time (i.e., virtually all 5th-graders in transparent orthographies can master the GPC-rules; Jiménez González and Hernández Valle, 2000; Burani et al., 2002), and the reading experience favors the development of orthographic-lexical knowledge (e.g., Carrillo et al., 2013). As a matter of fact, there is an early ceiling effect on PWR in Spanish. The performance of the children that participated in the validation of PROLEC-R (Cuetos et al., 2007) showed 95% of mean accuracy in PWR at 5th and 6th grades. This accuracy rate is equivalent to that found in Spanish adult healthy population, with 96% of mean accuracy (Cuetos and González-Nosti, 2009).

From these results, we can conclude that (1) both reading effects observed and reading processes inferred in opaque languages, as English, cannot be directly generalized to other transparent languages, as Spanish; (2) a simple task as PWR, which only involves the GPC process, is unlikely to adequately estimate the complexity of the cognitive skills that comprise the construct of intelligence quotient (e.g., the structure of the well-known WISC-IV intelligence test is composed of working memory, verbal comprehension, perceptual reasoning, and processing speed; Wechsler, 2003); and (3) the easiness of acquisition and mastering PWR in transparent orthographies, evidenced by a ceiling effect, casts doubts on its discriminant power.

## PWR and dyslexia

More support for the rationale of using PWR as estimate of PI presumably comes from the PWR performance shown by brain damaged or cognitive impaired patients. Del Pino et al. (2018) stated that several studies had criticized the use of the vocabulary test to estimate PI “…because vocabulary is known to decline with aging and is sensitive to brain damage” (p. 2). However, there is extensive literature that describes cases of acquired dyslexia after brain injury (Newcombe and Marshall, 1981). If vocabulary tests are not recommended to estimate PI because of their sensitivity to brain damage, PWR should not be employed in this quality either, at least for patients with possible damage in the left temporal and parietal areas. Moreover, we should not obviate that developmental dyslexia is characterized by specific difficulties with PWR (Suárez-Coalla and Cuetos, 2015); and, importantly, that this disorder has a considerable population prevalence of 10% (Jiménez et al., 2009; López-Escribano et al., 2018; Wagner et al., 2020). On the other hand, Del Pino et al. pointed out that reading ability, when automatized, is "highly resistant to cognitive impairment” (p. 2). Regarding this statement, evidence shows that word reading is more resistant to cognitive impairment in comparison to PWR, particularly when the pseudowords are not readable by lexical analogy but by graphemic-phonemic decoding (see the above-described results by Friedman et al., 1992 and by Patterson et al., 1994).

Another insight comes from the current definition of dyslexia by the International Dyslexia Association: “a specific learning disability that is neurobiological in origin. It is characterized by difficulties with accurate and/or fluent word recognition and by poor spelling and decoding abilities […] result from a deficit in the phonological component of language that is often unexpected about other cognitive abilities […].” (Lyon, 1995; Lyon and Shaywitz, 2003). The unexpectedness refers specifically to a discrepancy between expectations based on IQ and the actual reading achievement. There has been a debate for decades about whether to consider that discrepancy as a diagnostic criterion, and the debate has finally led to a consensus about its little usefulness for both researchers and clinicals (Siegel, 1989; Lyon and Shaywitz, 2003), precisely because of the weakness of the relationship between IQ and reading achievement (Stanovich, 2005). There is also a common consensus that developmental dyslexia is caused by a deficit in phonological processing, which is ubiquitous in all dyslexia subtypes and all alphabetical orthographies (Stanovich, 1988). The underlying basis for this consensus is that the phonological deficit is independent of IQ and, consequently, there is no reason to distinguish between learning-disabled (i.e., whit low IQ) poor readers and non-learning-disabled (i.e., with normal or high IQ) poor readers (Aaron, 1997). Thus, a great number of current investigations on dyslexia and reading assumes independence between IQ and reading achievement, at least when their focus is on the first steps in the reading process.

## Conclusion

In summary, for some reason, it has been assumed without sufficient evidence that PWR performance is an adequate estimate of premorbid PI. Furthermore, there is some reasonable evidence suggesting that such an assumption is unlikely to be true, at least in Spanish and in other transparent orthographies.

## Author contributions

All authors listed have made a substantial, direct, and intellectual contribution to the work and approved it for publication.
